# The Ambivalent Role of miRNAs in Carcinogenesis: Involvement in Renal Cell Carcinoma and Their Clinical Applications

**DOI:** 10.3390/ph14040322

**Published:** 2021-04-02

**Authors:** Federica Spadaccino, Margherita Gigante, Giuseppe Stefano Netti, Maria Teresa Rocchetti, Rossana Franzin, Loreto Gesualdo, Giuseppe Castellano, Giovanni Stallone, Elena Ranieri

**Affiliations:** 1 Clinical Pathology Unit, Center of Molecular Medicine, Department of Medical and Surgical Sciences, University of Foggia, 71122 Foggia, Italy; federica.spadaccino@unifg.it (F.S.); giuseppestefano.netti@unifg.it (G.S.N.); elena.ranieri@unifg.it (E.R.); 2 Department of Clinical and Experimental Medicine, University of Foggia, 71122 Foggia, Italy; mariateresa.rocchetti@unifg.it; 3 Nephrology, Dialysis and Transplantation Unit, Department of Emergency and Organ Transplantation, University of Bari, 70124 Bari, Italy; rossana.franzin@uniba.it (R.F.); loreto.gesualdo@uniba.it (L.G.); 4 Nephrology Dialysis and Transplantation Unit, Department of Medical and Surgical Sciences, University of Foggia, 71122 Foggia, Italy; giuseppe.castellano@unifg.it (G.C.); giovanni.stallone@unifg.it (G.S.)

**Keywords:** miRNAs, cancer, renal cell carcinoma, biomarkers

## Abstract

The analysis of microRNA (miRNAs), small, non-coding endogenous RNA, plays a crucial role in oncology. These short regulatory sequences, acting on thousands of messenger RNAs (mRNAs), modulate gene expression at the transcriptional and post-transcriptional level leading to translational repression or degradation of target molecules. Although their function is required for several physiological processes, such as proliferation, apoptosis and cell differentiation, miRNAs are also responsible for development and/or progression of several cancers, since they may interact with classical tumor pathways. In this review, we highlight recent advances in deregulated miRNAs in cancer focusing on renal cell carcinoma (RCC) and provide an overview of the potential use of miRNA in their clinical settings, such as diagnostic and prognostic markers.

## 1. Regulation of miRNAs and Their Ambivalent Role in Cancer

**Figure 1:**
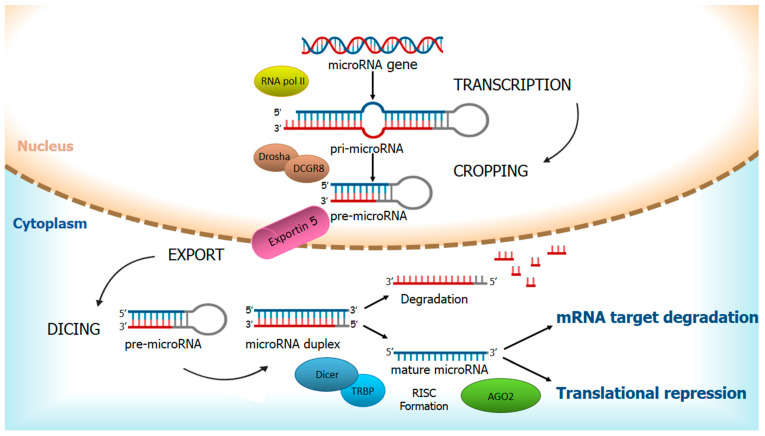
Biogenesis of microRNAs. The synthesis of primary-miRNA (pri-miRNA) by RNA polymerase II occurs in the nucleus. The following maturation involves three steps: cropping, in which pri-miRNA is processed by Microprocessor Complex, consisting of Drosha and DCGR8, to form a hairpin-like structure called precursor-miRNA (pre-miRNA); export, in which pre-miRNA is bound to Exportin 5 and transported into cytoplasm and dicing that leads to the generation of mature miRNA duplex. When the miRNA duplex is unwound, the passenger strand is degraded, and the leading strand is associated with a transactivation-responsive RNA-binding protein (TRBP) and Argonaute 2 (AGO 2) to form the RNA-induced silencing complex (RISC). The mechanism of action of RISC is based on the perfect or near-perfect complementarity to the 3′ UTR region of mRNA target resulting in a fine post-transcriptional regulation.

Overall, miRNAs are highly conserved among species and stand for 1% of the human genome [1]. The biogenesis of miRNAs is a multistep process that includes cytoplasmic and nuclear phases in which a long double-stranded RNA is progressively converted into a small single-stranded effector RNA (Figure 1).

In the nucleus, RNA polymerase II transcribes the first long primary strand of up to 10 kb, called primary-miRNA (pri-miRNA). Three more steps are required for complete maturation: *cropping*, in which Microprocessor Complex, consisting of Drosha, nuclear RNAse III, and DCGR8, double-stranded RNA binding protein, cleave pri-miRNA in a precursor-miRNA (pre-miRNA) to form a hairpin-like structure of about 70 bases [2]; *export*, in which pre-miRNA is bound to Exportin 5 and transported into the cytoplasm; and *dicing* in which pre-miRNA is processed by Dicer, cytoplasmic RNase III, to yield a 22–25 nucleotides mature miRNA duplex [3].

MiRNA duplex is then unwound, bringing on the degradation of the passenger strand and the binding of the leading strand to transactivation-responsive RNA-binding protein (TRBP) and Argonaute 2 (AGO 2) to form the RNA-induced silencing complex (RISC) [4]. Nonetheless, it is now well known that functional miRNAs can derive from both the 5′and 3′ arms of the duplex precursor (miRNA-5p and -3p, respectively), albeit targeting different sequences [5].

RISC binds to the 3′ UTR region on mRNA target and, depending on the perfect or near-perfect complementarity of this interaction, can induce fine post-transcriptional regulation resulting in mRNA degradation or translational repression.

Furthermore, a single miRNA can target different mRNAs, while different miRNAs can target the same mRNA. For this reason, the biological role of miRNAs is associated with the role of their target mRNAs. These observations well convey the idea of the regulatory potential of miRNAs in cellular processes. MiRNAs, in turn, are also regulated at both transcriptional and post-transcriptional levels with changes that may occur in the promoter of miRNA genes or in post-transcriptional factors, such as Drosha, Dicer, or Ago 2 protein, with consequent interference on miRNA processing and stability [6,7].

Cancer cells present abnormal proliferative ability with the potential to escape immune response and apoptosis and to sustain invasion, metastasis, and angiogenesis.

Since a single miRNA can positively or negatively modulate numerous target mRNAs, resulting in impaired gene expression, alterations in the expression of miRNAs have been reported as one of the pivotal mechanisms responsible for tumor onset and development.

In fact, germline and somatic mutations in the genes encoding miRNAs-processing enzymes have been identified in several malignant tumors such as pleuropulmonary blastoma, cystic nephroma and ovarian Sertoli-Leydig-type tumors [8,9,10]. Furthermore, more than 50% of human miRNAs are located in chromosomal regions that exhibit DNA amplification, deletions, or translocations during tumor development [11].

Like several regulatory genes, miRNAs also exhibit tissue-specific expression. Therefore, significant alterations of miRNAs expression levels in tumor tissue may be consequent to processes more directly involved in tumorigenesis, rather than being directly associated with cells neoplastic transformation [12]. Moreover, miRNAs may be crucial for the growth and maintenance of some cancer cells due to the phenomenon of oncogene addiction [13].

**Figure 2:**
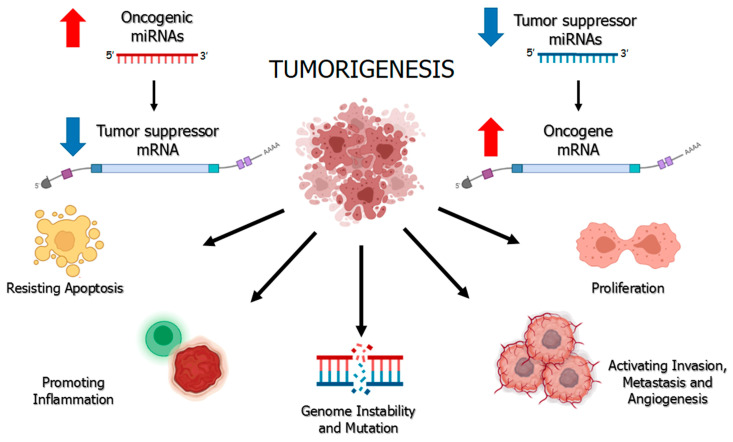
Aberrant expression of microRNAs in the tumor microenvironment. The upregulation of oncogenic miRNAs (oncomiRs), on the left, and the downregulation of the tumor suppressor miRNAs (TS-miR), on the right, have been shown to contribute to tumor development mechanisms by targeting tumor suppressor and oncogene mRNAs, respectively.

Tumorigenesis has always been considered due to deregulation of oncogenes and/or tumor suppressors due to genetic mutations (point mutations, insertions/deletions, chromosomal rearrangements) or epigenetic modifications. The discovery of miRNAs led to a better understanding of tumor development mechanisms. It is now well known that some miRNAs, highly expressed in cancer cells, play a pivotal role in carcinogenesis and act as oncogenes, increasing cell proliferation and decreasing apoptosis, or acting as tumor suppressors with opposite effects. The ambivalent role of miRNA is illustrated in Figure 2.

Oncogenic miRNAs (oncomiRs) are commonly overexpressed in cancerous tissues and are likely associated with the deregulation of tumor suppressor genes. For instance, solid tumors and diffuse large B-cell lymphoma present overexpression of stem-loop miR-17–92 cluster that impairs apoptosis mechanisms and inhibits cancer cell death [14].

Conversely, the low expression of tumor suppressor miRNAs (TS-miR) compared to their physiological condition causes overexpression of a target molecule, which, if it is an oncogene, will be directly associated with tumor growth. Common suppressor miRNAs are represented by miR-34a, whose reduced expression is linked to lung, pancreatic and breast cancer development; miR-15a and miR-16-1, which target antiapoptotic protein BCL2 and are deleted in chronic lymphocytic leukemia; mir-let-7 family that inhibits RAS oncogene and is poorly expressed in tumor tissues [15,16,17].

Regardless of the mechanism, the overall effect in case of activation or inactivation of a miRNA will be the under- or overexpression, respectively, of its target genes. It is the function performed by the target transcripts; therefore, that determines whether a miRNA is to be considered as oncogene or tumor suppressor [18].

As evidence, there are some oncomiR whose effect varies according to the mRNA they target and to the context in which they are found. For instance, miR-105 acts as an oncogene by promoting cell proliferation, metastasis and epithelial–mesenchymal transition (EMT) in colorectal, gastric and triple-negative breast cancer or as a tumor suppressor by inhibiting tumorigenesis in glioma, hepatocellular, prostate and non-small-cell lung (NSCL) cancer [19].

Similarly, miR-125b acts as an oncogene in hematologic neoplasms and as a tumor suppressor in several solid tumors, by targeting pro/antiapoptotic factors and metastasis promoters/inhibitors.

An ambivalent role is also played by miR-155, commonly considered as one of the most relevant oncogenes in hematologic malignancies but with a role of tumor suppressor in other cancer types [18].

## 2. miRNAs as Specific Noninvasive Biomarkers to Establish Diagnosis and Prognosis in Patients with Cancer

In the last few decades, cancer studies focused on the research of noninvasive markers to develop rapid and safe diagnostic protocols for cancer patients screening, therapy response monitoring, and disease recurrence evaluation. Experimental evidence has shown that miRNAs could represent valid diagnostic and prognostic noninvasive markers in human cancer since they result differentially expressed in tumor tissue when compared with normal counterparts, but also between primary and metastatic tumors.

The small size of these RNAs allows them to be more stable than long mRNA molecules and to be detected from fixed tissues or biological fluids. Several normal and cancer cell types can release miRNAs into the extracellular space.

Circulating miRNAs (C-miRNAs) can therefore be detected in biological fluids, including plasma, serum, saliva, urine and others. Recently, the presence of miRNAs in biological fluids has been observed in various cancers such as colon, prostate, breast, lung, ovary and other cancer types [20]. The characteristic tumor-specific origin suggests the possibility of using them as noninvasive discriminating markers for the analysis of tumor phenotype, in terms of diagnosis and prognosis [21].

C-miRNAs can interact with numerous cellular pathways and regulate gene expression of many recipient cells. This regulatory mechanism is allowed by the surprising characteristic of these miRNAs to be very stable in biological fluids since they can be released via extracellular vesicles, such as exosomes, microvesicles and apoptotic bodies or complexed to AG02 protein or high-density lipoproteins, being therefore resistant to RNAse digestion [22,23,24].

C-miRNAs can be thought as liquid biopsies that offer a promising prospect for noninvasive evaluation of the tumor mass. In fact, the term “liquid biopsy” refers to the use of biological fluids that trace the “molecular signature” released by cancer cells. From this, it follows that C-miRNAs are a valuable resource to support clinicians and researchers to expand the panel of tumor markers available for early diagnosis and prognosis of many cancer types.

However, even if C-miRNAs have been reported to be highly tumor sensitive and specific, currently, there are no miRNAs acclaimed for application in routine clinical practice. The combined use of several innovative markers, such as miRNAs panel, associated with the evaluation of classical tumor markers and common screening strategies is therefore highly recommended.

Moreover, a steadily growing number of studies suggest that miRNAs present a broad spectrum of potential clinical applications in cancer management, serving as diagnostic and prognostic tools. The specific miRNAs signature has been reported to allow different cancer types to be sensibly discriminated and primary or metastatic cancer to be identified according to miRNA tissue expression profile [25,26].

A specific 24 C-miRNAs profile was identified in the plasma of non-small cell lung cancer (NSCLC) patients with the ability to recognize early-stage cancer condition [27]. A recent study examined 2588 miRNAs profiles setting up a diagnostic model in which serum expression levels of miR-1268b and miR-6075 displayed a correlation with TNM staging [28].

Another study reported a panel of 34 C-miRNAs as able to predict prostate cancer development in patients who underwent radical prostatectomy, with miR-222 and miR-125b highly specific indicators of cancer progression risk [29].

Similarly, in a panel of 5 miRNAs that classify triple-negative breast cancer (TNBC) from non-TNBC, miR-199a-5p represent a highly specific miRNA for cancer staging [30].

Recently, bioinformatics approaches led to define a novel miRNA signature for the diagnosis of pancreatic cancer with the advantage of providing potential biomarkers [31].

MiRNA signature is also useful to estimate the patient prognosis. A panel of 84 C-miRNAs was evaluated in NSCLC patients and relative healthy controls, highlighting miR-23b-3p, miR-10b-3p, and miR-21-5p as indicators of poor overall survival [32].

A miRNA microarray analysis conducted on colorectal cancer (CRC) patients, identified miR-21-5p as the most expressed in exosomes, primary tumor tissues and liver metastasis tissue and as an independent prognostic factor for overall survival and Disease-free survival (DFS) in CRC patients [33]. In addition, Yang et al. uncovered five-miRNAs signature as a new predictive model of CRC prognosis [34].

Similarly, Hua et al. showed downregulation of miR-373 expression in pancreatic cancer patients and its correlation to worse clinical parameters and shorter overall survival [35].

Two different four-biomarker miRNAs signatures were found to act as an independent prognostic factor for overall survival of TNBC and cervical cancer patients [36,37].

Moreover, meta-analysis studies revealed that high expression of miR-195 and miR-130 family might predict poor prognosis and survival in population with cancer [38,39].

Taken together, these studies suggest the important role played by miRNAs as prognostic tools for clinical application.

## 3. miRNAs in Cancer Therapy

At present, chemotherapy, radiotherapy, and surgery represent the most common therapeutic options in patients with cancer. However, not all patients respond well to treatment and not all the cancers are sensitive to common protocols. A crucial goal of recent studies focused on miRNA characterization is to find potential biomarkers able to increase sensitivity to chemio-radiotherapy, predicting patient’s response and resistance to targeted therapy. As previously reported, miRNAs expression may modify the expression of oncogenic or anti-oncogenic protein as well as it is essential for cancer development and metastasis, proving that miRNAs may be used as therapeutic tools in cancer treatments.

miRNA inhibition therapy decreases the expression of oncomiRs that are frequently overexpressed in human cancers by treatment with anti-miR oligonucleotides (AMOs), locked nucleic acid (LNA), anti-miRNAs, antagomirs, miRNA sponges, miRNA masks and small molecule inhibitors of miRNAs. Alternatively, normal expression of tumor suppressor-miRNAs involves miRNA restoration therapy by the use of small molecules, synthetic miRNA mimics, and DNA plasmids technologies [40,41,42]. The role of miRNA, as tumor suppressor miRs or oncomiRs, has been investigated in various cancers, such as lung cancer, breast cancer, colon cancer, hepatocellular carcinoma, gastric cancer, etc. In this review, we mainly focus on the effects of miRNAs in renal cell carcinoma (RCC). The contribution of miRNA to RCC will be highlighted in the following section.

## 4. Renal Cell Carcinoma

Renal cell carcinoma (RCC) is the most common type of renal neoplasia, and it accounts for about 3% of all adult malignancies in western countries [43]. The RCC is mostly a tumor of older adults with the peak incidence occurring between 60 and 70 years of age and it is higher in the male gender and in more developed countries. Its overall mortality rate reaches 2.2 cases/100.000 persons/year [44].

Due to usually asymptomatic clinical course, the diagnosis of most cases of renal cancers is often accidental, following diagnostic tests performed for other clinical conditions and not rarely shows neoplasms in advanced clinical stage [45].

Adenocarcinoma is accountable for about 90% of RCC cases and shows three main variants: clear cell renal carcinoma (ccRCC), papillary (pRCC) and chromofobe (chRCC) variant [46].

ccRCC is the most frequently diagnosed form and, based on its natural metastatic tendency, is also the most clinically severe and it is responsible for about 90% of RCC deaths [47,48,49].

RCC is chemo- and radioresistant neoplasia; therefore, the current therapeutic strategies are based on the surgical approach (radical and/or partial nephrectomy) with a 5-year survival rate estimated to be around 70–80% for patients with localized cancer compared to 10% for metastatic forms [50,51,52].

Recently, a growing number of studies have been focused on the pathogenetic role of miRNAs to better understand the mechanisms underlying neoplastic development and, consequently, to identify new molecular and therapeutic targets.

## 5. Deregulated miRNAs in RCC

**Figure 3:**
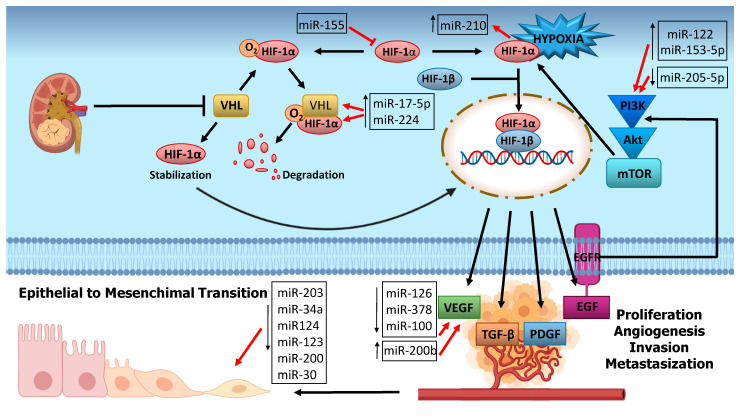
Involvement of the significantly dysregulated microRNAs in renal cancer signaling pathways. Red arrows show the contribution of miRNAs to the development of RCC. As illustrated, miR-17-5p, miR-224 and miR-210 contribute to tumor growth by targeting the pathway of HIF-1α (Hypoxia inducible factor alpha), and its target, in normoxia and hypoxia conditions. Conversely, miR-155 impairs HIF-1α expression. The downregulation of tumor suppressor miR-126, miR-378, miR-100 and the upregulation of oncogenic miR-200b promote angiogenesis. Tumor growth, proliferation and metastasis depend on dysregulated miR-122, miR-153-5p and miR-205-5p which impact PI3k/Akt/mTOR (PI3K, phosphatidylinositol-3-kinase; AKT, protein kinase B; mTOR, mammalian target of rapamycin) signaling pathway and on the downregulation of miRNAs panel that promote epithelial to mesenchymal transition of renal cancer cells.

Different studies on miRNAs expression profiles in RCC have identified deregulated miRNAs panels, able to discriminate tumor from normal condition, malignant from benign neoplasms and metastatic from localized forms (Figure 3).

First studies on miRNA analysis in RCC showed an upregulation of oncomiRs, such as miR-16, -21, -34a, -452, -224, -155, and -210 and a downregulation of TS-miR, such as miR-141, -149, -200b, -363, -429, -200c, -514, and -141 [53,54].

Recently, Lokeshwar et al. identified miR-21, miR-142-3p, miR142-5p, miR-150, and miR-155 as upregulated and miR-192 and miR-194 as downregulated in RCC [55]. MiR-21 is upregulated in various cancers, its overexpression is useful to discriminate ccRCC from pRCC subtypes and it correlates with the progression of stage and grade of tumors [56]. MiR-21 correlates with growth, apoptosis modulation and cell cycle progression of RCC cells [57]. In addition, Wang and colleagues demonstrate the association between high expression levels of miR-21 and the major invasive ability of RCC cell lines and primary cancer cells compared to human renal tubular epithelial (HK2) cells [58].

As an oncogene, miR-23a-5p has been highly expressed in RCC tissues when compared to normal samples, and it has been correlated with the development and proliferation of RCC [59]. In acute myeloid leukemia, Hatzl et al. found a correlation between the increased expression of miR-23a and the decreased expression of RAF kinase inhibitor protein (RKIP), a regulator of intracellular signaling involved in anti-metastatic process in several malignancies [60]. Interestingly, in our study, we found RKIP downregulated in RCC patients, although no studies have yet evaluated its correlation with miR-23a in RCC [61].

Similarly, miR-155 is involved in numerous cancer types and its upregulation in ccRCC correlates with an increase in proliferation and invasion by targeting Forkhead box O3a (FOXO3a) and E2F transcription factor 2 (E2F2) [62,63].

On the counterpart, among downregulated miRNAs, miR-200b is associated with clinical overall survival and M stage of RCC by targeting Laminin subunit alpha 4 (LAMA4), involved in angiogenesis and tumor metastasis, while miR-508 is associated with proliferation and invasion of RCC by targeting Zinc finger E-box-binding homeobox 1 (ZEB1) [64,65].

Taken together, these studies revealed the complicate and intricate role that miRNAs play in RCC carcinogenesis, aspects, which will be explored in the next sections.

## 6. miRNA and RCC Carcinogenesis

### 6.1. Hypoxia Related miRNAs

Most forms of RCC are associated with loss of function mutations occurring in VHL (Von Hippel-Lindau Tumor Suppressor) gene, which normally encodes for pVHL protein with consequent hypoxia-induced factor 1 (HIF-1) degradation. Hypoxia and VHL mutations subvert this mechanism and stabilize HIF-1 expression, which is responsible for many downstream effects, such as angiogenesis, tumor growth and metastatization [66].

Several studies have reported that VHL and miRNAs regulate each other in either a HIF-dependent or HIF-independent manner in RCC. Although VHL and HIF are key molecules, the action of miRNAs, also affects HIF target genes such as vascular endothelial growth factor (VEGF) platelet-derived growth factor (PDGF), epidermal growth factor receptor (EGFR), transforming growth factor β (TGF-β) and HIF-correlated signaling pathways such as KRAS and the mitogen-activated protein kinase (MAPK) pathway, phosphatidylinositol 3-kinase (PI3K) and Akt pathway with mammalian target of rapamycin (mTOR) as its target.

The above-mentioned miR-155 is also involved in this pathway and it could represent a promising therapeutic target [67].

Several studies reported HIF1 and VHL to be direct targets of miR-17-5p, which appear dysregulated in RCC and induces proliferation and migration in various cancer types [68].

Respectively, VHL and HIF1 are the direct and indirect targets of miR-224, which is upregulated in RCC and displays an oncogenic role in many cancer types [69].

MiR-210 represents the main hypoxia-induced miRNA and correlates with negative clinical outcome in most solid tumors. MiR-210 has been shown to be upregulated in RCC and to correlate with a good prognosis [70].

### 6.2. Angiogenesis-Related miRNA

Angiogenesis represents one of the pivotal mechanisms responsible for developing RCC. It can promote abundant vascularization leading to cell proliferation and metastasization. The major role in the angiogenic process is played by VEGF and its receptors whose expressions correlate with tumor size and stage and a poor prognosis [71,72].

Since VEGF is a target of HIF, the same miRNAs can impact both factors. This is the case of miR-17-5p that has shown an inverse correlation with VEGF expression in RCC patient samples [73].

Li et al. analyzed and compared the expression of angiogenesis-related miRNAs in tissues of RCC patients, showing that patients with early-stage disease expressed lower levels of miR-126 and miR-378 in tumors compared with normal renal tissues, whereas higher levels of expression of these miRNAs were found in later stages [74]. miR-378, in association with miR-100, was also evaluated by Chen S.C. et al. who found a lower expression in metastatic ccRCC when compared to non-metastatic patients [75].

de Càssia Oliveira et al. has observed an increase of miR-200b, which is independently correlated with high-risk tumors similar to microvascular invasion, tumor size and high Fuhrman’s grade in ccRCC samples. A decrease of miR-126 was also observed in correlation with VEGFA (vascular endothelial growth factor) gene overexpression, supporting the hypothesis that miR-126 acts as oncosuppressor gene [76].

Fuertes et al. evaluated miRNAs expression profiles to better characterize RCC subtypes and found an interesting miRNAs pro-angiogenesis signature, represented by miR-185, miR-126, and miR-130 that could define a potential proangiogenic cluster to select patients for antiangiogenic therapy [77].

### 6.3. PI3K/Akt Pathway Related miRNA

Hypoxia and angiogenesis factors affect many signaling pathways, among which EGFR-PI3K-Akt is of great relevance. This pathway regulates several cellular processes. Elevated Akt and its target levels correlate with tumor proliferation and during the years, many studies have been focused on it to define possible targeted therapies. One of the major effects is the activation and phosphorylation of mammalian target of rapamycin (mTOR), involved in several molecular processes, that correlates with tumor progression and invasion. Mutations in PI3K/Akt/mTOR signaling occur regularly in RCC. Inhibitors of mTOR, Everolimus and Temsirolimus, have shown efficacy in metastatic RCC treatment [78,79].

Raush et al. recently observed that mTOR show higher expression in metastatic than primary tissues of RCC and that elevated phosphorylation level of mTOR is significantly related to an advanced tumor stage with a high Fuhrman grade and unfavorable outcome [80].

Among miRNAs, miR-122 has been upregulated in renal cancer cells and may play a key role in promoting tumor proliferation by activating PI3K/Akt signaling pathway [81].

Decreased expression of miR-205-5p in RCC tissues compared to normal tissues was found in association with poor clinical outcomes. In addition, the authors of this study observed that miR-205-5p directly targeted the 3′ UTR of VEGFA, concluding that miR-205-5p acts as a tumor suppressor by targeting VEGFA and PI3K/Akt signaling [82].

A recent study identified miR-153-5p as responsible for ccRCC occurrence and progression. The upregulation of miR-153-5p and the relative downregulation of its target AGO1 correlate with increased proliferation and metastatization of ccRCC by activating PI3K/Akt signaling [83].

### 6.4. EMT-Related miRNAs

RCC, especially the clear cell variant, has a natural metastatic tendency. Tumor metastases are not only correlated with disease severity but also to therapy failure and cancer-related death.

The epithelial–mesenchymal transition (EMT) is a process by which cells, losing their epithelial features and getting a mesenchymal phenotype, contribute to the metastasis process.

The PI3K/Akt pathway impairs cellular growth, proliferation, differentiation and angiogenesis and for these reasons, may induce EMT in RCC and all over the cancer types.

A decreased expression of miR-203 was linked to the upregulation of caveolin-1 (CAV1), a structural protein involved in the AKT/mTOR signaling pathway in advanced RCC. The overexpression of miR-203 performed in in vitro experiments, alters CAV1 expression, which in turn inhibits the EMT process, suggesting a suitable role as a therapeutic target in RCC [84].

The overexpression of miR-34a promoted EMT in cultured HK2 cells, which correlates the downregulation of its target Klotho, inhibitor of renal fibrosis and oncosuppressor factor in RCC [85,86].

A recent study revealed that miR-124 and miR-203 were downregulated in RCC tissues and this was consistent with a decrease in epithelial marker expression and an increase in mesenchymal markers. In addition, miR-124/-302 combined action could regulate Zin finger E-box-binding homeobox 2 (ZEB2), a key activator of EMT [87].

Mlcochova et al. conducted a study on miRNA expression to evaluate the prognostic significance of EMT in RCC. MiR-200 and miR-30 families were the most deregulated EMT-related miRNAs and they were also associated with TNM stage, Fuhrman grade and OS of ccRCC patients [88].

## 7. MiRNA as a Biomarker of RCC

Currently, several predictive parameters have been established for managing RCC, including tumor staging (TNM) and grading (Fuhrman), histological subtype and other clinical-laboratory parameters.

Many molecular pathogenic targets such as HIF, VEGF, carbonic anhydrase IX (CaIX), phosphatase and tensin homolog (PTEN), C-reactive protein (CRP), Erythropoietin (EPO), E-cadherin, C-X-C chemokine receptor type 4 (CXCR4), CD44, PAX8 and PAX2, Ki67, p21, p53, and other potential RCC biomarkers have been investigated. However, for a more accurate evaluation and in line with the necessity to develop personalized therapeutic protocols, several studies have been oriented towards the research and identification of new biomarkers in order to reinforce the current diagnostic and prognostic tools [89,90,91,92,93].

Since RCC does not show signs of disease during carcinogenesis, several studies have been focused on the research of tissue, serum and urinary biomarkers. Table 1 includes a list of miRNA used as biomarkers to establish a diagnosis and a prognosis in RCC patients.

miR-378 and miR-451 were found differentially expressed in the serum of RCC patients and were related to the RCC diagnosis [94]. It has been reported that miR-129-3p expression can discriminate between malignant from benign renal tumors and that its low tissue level is associated with anti-metastatic property [95].

Toraih et al. indicated a correlation between the expression of miR-34a and its target genes in RCC tissues, such as MET, E2F3, TP53 and SOX2, displaying a potential role in RCC tumorigenesis and progression [96]. Another study revealed serum miR-21 and miR-106 levels significantly higher in RCC patients when compared with healthy donors and decreased after surgical treatment [97].

Wang et al. have detected a decreased expression of miR-200a in serum and urinary samples of RCC patients compared to controls [98].

A recent study identifies miR-122, miR-1721, and miR-15b as a potential urinary biomarker of RCC with higher sensitivity and specificity [99].

An analysis conducted on plasma-derived exosomes has detected about 245 miRNAs differentially expressed among RCC patients and controls. In particular, miR-92a-1-5p, miR-149-3p, and miR-424-3p have been identified as the most accurate for the diagnosis of RCC [100].

Lou et al. have found that plasma miR-144-3p levels are upregulated in ccRCC with the advanced pT stage compared with healthy controls and significantly decrease after surgical treatment [101].

An increase in serum miR-122-5p and miR-206 levels is also associated with adverse clinical parameters, shorter OS in ccRCC patients compared to healthy individuals [102].

The miRNA let-7 family is considered a tumor suppressor miRNA and it has been found downregulated in RCC and other types of tumor tissues. Fedorko et al. reported the all let-7 miRNAs are significantly upregulated in urine samples of RCC patients when compared to normal individuals, suggesting their role as a noninvasive biomarker of RCC [103]. MiR-122 and miR-30a correlate with ccRCC metastasis [104,105] and miR-34a, miR-141, and miR-1233 can contribute to the diagnosis of ccRCC [106].

Taken together, these studies show the potential role of miRNAs as biomarkers. However, more investigations are needed to better understand how to translate this information from research to clinic management to outline a specific signature that could allow us to distinguish healthy from RCC patients.

## 8. miRNAs in RCC Therapy and Future Application in Clinical Practice

**Figure 4:**
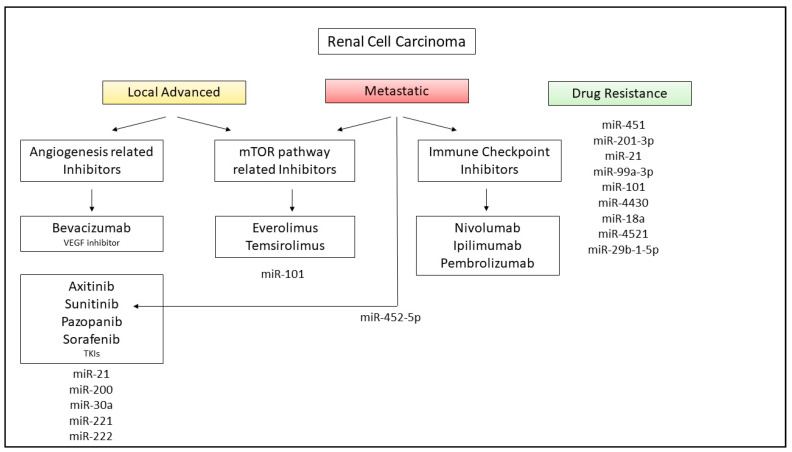
Involvement of miRNAs in RCC therapy. Targeted drugs commonly used for the clinical management of local advanced and metastatic RCC with the indication of miRNAs evaluated during or post-treatment.

The identification of therapeutic targets for cancer treatment is an ongoing growing field. Several miRNAs can increase or decrease the sensitivity to common therapeutic protocols (Figure 4).

Chemotherapy is the first-line approach after surgery and the main reason for chemotherapy failure is the development of multidrug resistance (MDR) to chemotherapy agents.

It has been reported that miR-451 reinforced drug resistance during chemotherapy while its inhibition improved drug susceptibility in ACHN cell lines [107]. Similarly, miR-210-3p significantly impairs the renal cell response and has been detected as differentially expressed among drug-resistant and drug-sensitive RCC cells [108]. Mir-21 acts as oncogenic miRNA leading to increased proliferation, invasion and anti-apoptotic signaling pathways in RCC and contributes to mediating RCC chemoresistance. miR-21 silencing is a promising option to improve chemotherapy response and ease patient care [109].

The development of targeted immunotherapy and personalized protocols has certainly contributed to improving the therapeutic approach, providing a better outcome for patients with cancer.

Nonetheless, the success or failure of drug action depends not only on its affinity with the tumor but also on the modulation that the microenvironment exerts on the tumor to allow cancer cells to survive.

To date, two types of target drugs are promising in advanced ccRCC treatment: inhibitors of VEGF (bevacizumab) or angiogenesis tyrosine kinase inhibitors such as Axitinib, Sunitinib, Pazopanib and Sorafenib and inhibitors of the mTOR signaling pathway (Everolimus and Temsirolimus) [110].

Bevacizumab is a recombinant monoclonal antibody that neutralizes the activity of VEGF, the major responsible factor inducing angiogenesis. Axitinib is also a drug aimed to inhibit tumor angiogenesis by targeting VEGF and PDGF receptors. Sunitinib, Sorafenib and Pazopanib are a low molecular weight TKI directed against PDGFRα e PDGFRβ and VEGFR1, VEGFR2 e VEGFR3.

One of the main targets of antineoplastic therapy in RCC patients is the mTOR serine/threonine kinase, which forms the catalytic subunit of the complex molecular mTORC1 and is involved in numerous processes responsible for tumor progression. The finding of phosphorylated mTOR on the Ser2448 residue represents a specific marker of activation of its pathway in ccRCC, leading to the activation of downstream target factors, such as S6K and 4E-BP1.

Everolimus and Temsirolimus belong to the mTOR inhibitor group. By analysing the expression levels of phospho-mTOR and phospho-S6RP, it is possible to predict the efficacy of treatment with these drugs, as higher levels of these proteins are associated with longer survival in patients with metastatic RCC [111].

Also, Metformin, a common drug used in diabetes, suppresses effects on RCC proliferation.

Metformin could induce G0/G1 cell cycle arrest and impair RCC growth in vitro and in vivo by inhibiting the AKT/mTOR pathway, while Sunitinib and Sorafenib suppress cell proliferation and angiogenesis by targeting VEGF and PDGF receptors [112,113].

It has been reported that in RCC cells, Metformin can decrease miR-21 levels and increase PTEN expression, altering pAkt levels.

Sensitivity to Sorafenib was increased in association with miR-21 and miR-200 [114,115].

Zheng et al. has reported that miR-30a, inhibitors of autophagy, is downregulated in RCC cells and tissues in correlation to the upregulation of its target, Beclin-1. Exogenously expression of miR-30a increases Sorafenib-induced cytotoxicity, showing how miR-30a could be a novel potential therapeutic target for RCC [116]. Khella et al. provided evidence that miR-221 and miR-222, targeting VEGFR, are promising predictive markers for Sunitinib [117]. MiR-452-5p has been reported to be upregulated and associated with poor prognosis in RCC. Nonetheless, its expression is attenuated by Sunitinib, suggesting that miR-452-5p could be a new therapeutic tool for mRCC protocol [118]. Sunitinib is one of the most common targeted drugs for mRCC; however, RCC cells have shown molecular mechanisms of drug resistance.

MiR-99a-3p is downregulated in RCC cells and is involved in Sunitinib-resistant RCC [119]. Similarly, miR-101 has been downregulated in Sunitinib-treated RCC tissues and to be associated with the overexpression of UHRF1, contributing to drug resistance [120].

Yamaguchi et al. have analysed Sunitinib-resistant cells and identified seven miRNAs differentially expressed compared to control cells. Among these miRNAs, miR-4430 has been correlated with the PTEN and mTOR pathway; miR-18a-with HIF1A; miR-4521 with ZEB2 and the mTOR pathway; miR-29b-1-5p with the PI3K/Akt and HIF1 pathways. Taken together, these target genes or pathways could be involved in the mechanism responsible for Sunitinib resistance of RCC [121].

As mentioned above, the inhibition of PI3k/Akt/mTOR pathway may be a valid choice for mRCC treatment. Everolimus and Temsirolimus belong to the group of mTOR inhibitors and represent the first- and second-line settings for treating metastatic RCC [122].

MiR-101 is involved in several types of cancer development since it provides a downstream activation of mTOR signaling pathway, including mTORC2 and HIF-2α [123].

Nogueira et al. have observed that resistance to mTOR inhibitors is related to the overexpression of HIF-2α and that circulating levels of miR-101, being excreted by Everolimus-resistant cells, could improve anti-mTOR therapy response and predict resistance [124].

Among the immune checkpoint inhibitors, Nivolumab is a monoclonal antibody targeting Programmed Death-1 (PD-1) receptor on activated T lymphocytes, that inhibits its binding to the relative receptor expressed on tumoral cells, leading to the activation of the immune response against a tumor. Several combinations of antiangiogenic agents and immune checkpoint inhibitors are being studied and tested. The combination of Nivolumab with the Ipilimumab, an anti-CTLA4 monoclonal antibody, has been authorized in 2019 for the first-line treatment of RCC in patients who were not previously treated, at moderate or at high risk of worsening. The combination of these two drugs, compared to Sunitinib, improves PFS among patients [125]. A specific subset of miRNA expressed in peripheral lymphocytes was induced by Nivolumab treatment and may serve to guide clinical decisions in RCC patients [126].

In addition, Nivolumab leads to superior overall survival compared to Everolimus in patients failing one or two lines of VEGF-targeted therapy [127].

The combination of Pembrolizumab, an inhibitor of PD-1, and Axitinib in treatment-naïve patients with metastatic ccRCC across all risk groups demonstrated overall survival and overall relative risk benefits compared to sunitinib [128].

Considering the great potential of miRNAs, several clinical trials have been proposed [129]. A recent study reports that MRX34, a mimic of miR-34 encapsulated in a liposomal nanoparticle (LNPs), was the first miRNA developed by MiRNA Therapeutics to have been tested for the treatment of various cancer types, including RCC. Although the first results were encouraging, especially for patients affected by metastasized cancers, immune-related adverse responses led to the trial to be temporarily interrupted [130].

Despite recent advances, further studies are needed to expand the therapeutic strategies currently available.

## 9. Conclusions

MiRNAs play a role in almost all aspects of cancer biology and development and considering their multi-level involvement in the major cancer pathways; they are also promising therapeutic targets. RCC is still considered one of the most unfavorable neoplasms in terms of prognosis and it is difficult to diagnose. To date, there are no specific biomarkers validated for its early detection. The study of miRNAs could allow us to expand the spectrum of potential biomarkers to be set and used for the diagnosis and management of RCC. Thus, miRNAs could provide a new starting point at therapeutic level with an increase of new clinical trials and their translation into clinical practice would be of relevant benefit in this neoplasia.

## Figures and Tables

**Table 1:** miRNAs as biomarkers for RCC early detection and prognosis.

miRNAs as Biomarker in RCC	Source	Therapeutic Aim
miR-378, miR-451	Serum	Diagnosis
miR-129-3p	Tissue	Prevention of metastasis
miR-34a	Tissue	Proliferation
miR-21 and miR-106	Serum	Diagnosis and prognosis
miR-200a	Serum, Urine	Diagnosis
miR-122, miR-1721, miR-15b	Urine	Diagnosis
miR-92a-1-5p, miR-149-3p, miR-424-3p	Plasma-derived exosomes	Diagnosis
miR-144-3p	Plasma	Prognosis
miR-122-5p, miR-206	Serum	Prognosis
miR let-7	Urine	Diagnosis
miR-122, miR-30a	Tissue	Metastasis
miR-34a, miR-141, miR-1233	Tissue	Diagnosis
